# A Three-part Quality Improvement Initiative to Increase Patient Satisfaction and Reduce Appointment Time

**DOI:** 10.1097/pq9.0000000000000277

**Published:** 2020-03-09

**Authors:** Sujal Manohar, Crystal McLeod

**Affiliations:** 1 From the PediPlace Clinic Lewisville, Lewisville, Tex.

## Abstract

**Introduction:**

PediPlace, a busy pediatric not-for-profit clinic in Lewisville, Tex., struggles with prolonged patient wait times. This quality improvement (QI) project aimed to reduce appointment lengths and improve the patient experience.

**Methods:**

PediPlace introduced 3 interventions: improved patient tracking to measure timing on the electronic health record interface, elimination of script repetition between medical assistants and providers, and patient art kits.

**Results:**

From over 2,000 surveys indicated that patients were more satisfied; the percentage of negative survey comments decreased from 20% in 2017 and 2018 to under 10% in 2019. The overall rating of the clinic rose from 3.8 to 4.75 (on a scale of 1–5) after QI interventions as well. The number of patients not checked out declined from 49 errors in October–December 2018 to 25 errors in January–March 2019, indicating that this part of the initiative was initially successful. However, subsequent months experienced higher error rates. Analysis of over 20,000 appointments from June 2018 to July 2019 showed that appointment length did not change after interventions.

**Conclusions:**

There was no difference in appointment lengths between Hispanic/Latino patients and other patients, and an encouraging finding that suggests PediPlace providers are effective bilingual communicators. Furthermore, anecdotal evidence indicated that the art kits were positively received. The interventions appeared to improve the patient experience but did not impact appointment length. PediPlace plans to continue QI initiatives in the future.

## Abstract 2

Supplemental Digital Content is available in the text.

## INTRODUCTION

At PediPlace, a busy primary care not-for-profit pediatric clinic, extended wait times frustrated both patients and providers. Many patients spent over 2 hours at the clinic for a 15-minute appointment. A poor patient tracking system exacerbated this challenge, making it difficult to measure appointment length or to estimate workflow delays accurately. If a patient was not checked out in the system, it appeared as if they were in the clinic for several hours. This issue resulted in inaccurate data regarding appointment lengths. Furthermore, pediatric patients were often bored due to long waits.

Although it may be difficult to quantify the negative impact of prolonged wait times, previous studies show they worsen nearly every aspect of the patient experience. Longer wait time is negatively correlated not only with patient satisfaction but also with confidence in the provider and the perceived quality of care.^1,4^ Reduced wait times correspond to greater willingness to return and follow up with specialty care and improves pediatric patient health.^2^ Wait time also affects patient loyalty and the likelihood to recommend the clinic to others.^3^ Furthermore, related studies demonstrate that distractors in the waiting area improve the waiting experience for children.^5^ For example, art therapy programs are effective with other patient populations.^6^ For patients, their families, and providers, it is of utmost importance to increase efficiency and better the healthcare experience by reducing waits and providing engaging activities.

This project aimed to address these challenges within the context of PediPlace, a Texas not-for-profit pediatric clinic. The initiative utilized the Standards for Quality Reporting Excellence guidelines for quality improvement reporting.^7^

## SPECIFIC AIMS

The clinic intended to improve efficiency and satisfaction through a quality improvement (QI) project by decreasing total appointment length for sick-child visits to 45 minutes and well-child visits to 60 minutes. In July 2018, before the QI project began, sick-child visits took 1 hour and 10 minutes, and well-child visits took 1 hour and 30 minutes on average. PediPlace also aimed to improve patient satisfaction based on survey responses.

## METHODS

### Project Context

This QI project took place at PediPlace, a not-for-profit pediatric clinic with 2 main locations in Lewisville and Dallas, Tex. There is also a third school-based clinic at Central Elementary School in Lewisville. PediPlace conducted the initiative at the Lewisville clinic, as it is the busiest of the 3 locations.

As a nonprofit provider primarily serving the uninsured and Medicaid population, PediPlace must follow the Texas Medicaid policy. The Medicaid policy requires parents to change the child’s primary care provider on their insurance card via phone before the end of the visit to ensure that the visit is reimbursed to the correct office. This process includes providing a confirmation number to checkout staff. Though not considered a specific delay during the appointment, this process is time-consuming and adds to the entire appointment length. Texas Medicaid policy also requires the completion of developmental screening questions at each visit through the age of 60 months.

PediPlace sees between 70 and 100 patients daily for sick-child and well-child appointments. About 55% of the patient population identifies as Hispanic or Latino. Four full-time and 6 part-time providers see patients 1–3 days a week. Each provider sees 15–20 patients per day. The clinic has 18 patient examination rooms, 2 triage areas, separate sick and well side waiting rooms, and a front office with 2 check-ins and 2 checkout personnel. PediPlace has 9 medical assistant (MA) positions, 8 of which are currently filled. Each provider uses 2 examination rooms, with an additional shared overflow room. PediPlace uses the NextGen electronic health record (EHR) Interface (NextGen Healthcare, Irvine, Calif.).

### Standard Workflow

The front desk receptionist first registers patients when they arrive. The MA moves patients from the waiting area to a triage room to record vitals. The MA then guides patients to an examination room and completes the first portion of the examination that consists of teaching, recording health history, and/or completing vision and hearing screens. After a variable waiting period, the provider enters the examination room and completes the appointment. The MA then walks patients and their families back to the front desk to check out. MAs clean examination rooms between patients.

**Fig. 1.**
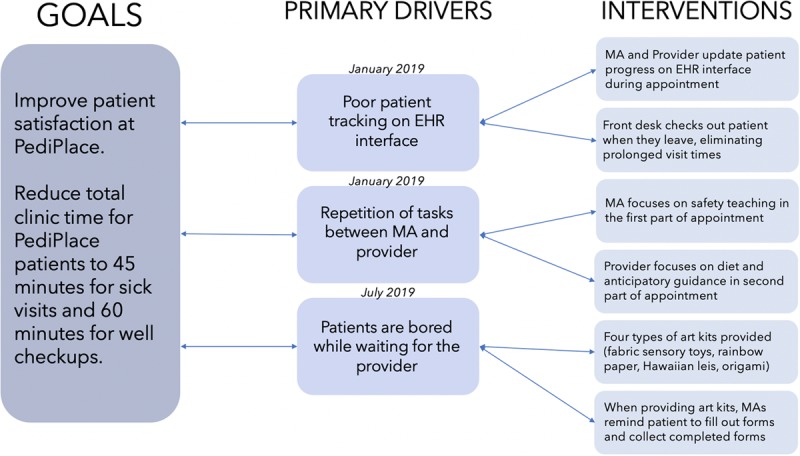
Summary driver diagram showing the 3 key drivers: inaccurate patient tracking, duplicative teaching information from both MAs and providers, and patient boredom during waits. The far-left outlines the overarching goals of the QI project. To the far-right are the interventions addressing each of the key drivers. PCP, primary care provider.

The time spent in the clinic depends on the appointment type. Sick and well visits are scheduled for 15 and 30 minutes, respectively. Often, appointments run longer than planned because parents bring up new issues while meeting the provider. Another potential source of delay is the failure of patients to check-in upon arrival, causing a longer initial wait and cascade of a longer appointment. Based on observation, PediPlace created a flow diagram with the approximate time spent on each part of the appointment and potential delay sources (Fig. 1).

### Interventions

**Fig. 2.**
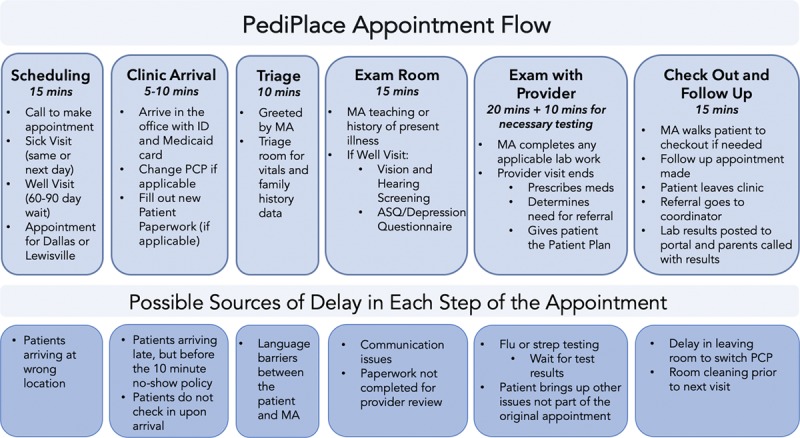
Appointment workflow diagram derived from observation outlining the main steps in each appointment at PediPlace andestimated time spent. The bottom boxes show potential sources of delay at each step.

PediPlace staff developed a key driver diagram and chose 3 QI interventions for this project based on clinic observations (Fig. 2). The drivers include inaccurate patient tracking, duplicative teaching information from both MAs and providers, and patient boredom during waits. Implementing the interventions in a stepwise approach was the best way to address these issues at PediPlace.

### Improving Patient Tracking in EHR (NextGen) Interface (implemented in January 2019)

To improve the accuracy of appointment tracking, all providers and staff began diligently monitoring appointment flow. MAs and providers updated the patient’s progress during each step of the appointment on the NextGen EHR Interface (NextGen Healthcare, Irvine, Calif.). This patient tracking piece was user dependent, and data collection contained only total appointment length time. Data collection did not include timing information for the subsections of the appointment. The front desk reminded patients leaving the clinic to check out, recording the ending time of the appointment accurately. MAs walked many patients to the front desk to facilitate the checkout process, particularly those who required follow-up appointments. This intervention provided more accurate data regarding total appointment length.

### Eliminating Repetition of Tasks Between MA and Provider (Implemented in January 2019)

**Fig. 3.**
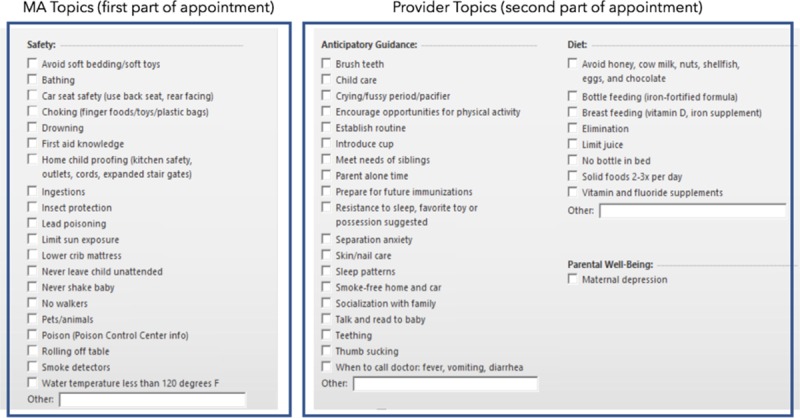
Example from EHR Interface showing the separate topics covered by MAs and providers.

Previously, at well-child visits, both providers and MAs discussed anticipatory guidance, diet, and safety information. The MA and provider teaching scripts were revised to enhance efficiency and reduce appointment length (Fig. 3). For well-child visits, MAs focused on safety teaching in the first part of the appointment. Following this, the provider gave the patient anticipatory guidance and diet information.

### Art Activity Kits in Waiting Rooms (Implemented in July 2019)

**Fig. 4.**
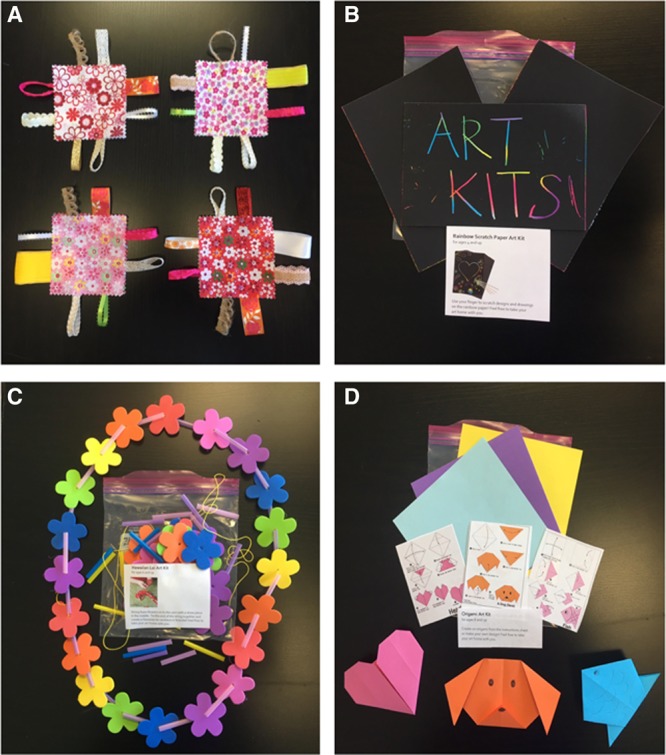
Art activity kit examples showing kits designed for different age groups. The kits included instruction sheets in both English and Spanish. A, Fabric sensory toys for ages 0–4. B, Rainbow scratch paper for ages 4 and up. C, Hawaiian lei art kit for ages 6 and up. D, Origami art kit for ages 8 and up.

Art kits were the third intervention as they have been utilized successfully in other in-patient settings.^8^ MAs offered art activity kits to pediatric patients while they waited for the provider (Fig. 4). MAs distributed these kits to patients in examination rooms when patients had been waiting for the provider for longer than 10 minutes. As part of this intervention, MAs started reminding parents to fill out necessary paperwork/questionnaires when providing the kits to patients. This change facilitated the completion of paperwork that the provider needed to review before entering the examination room. As incomplete paperwork can lead to delays, this reminder helped ensure all questionnaires were returned, scored, and reviewed by the provider before entering the examination room.

#### Measures

The initiative used a clinic-wide collaborative approach involving the front office staff, MAs, and provider teams. PediPlace implemented 3 interventions and measured the impact by tracking survey responses and total appointment length. The QI project examined the qualitative and quantitative aspects of patient experience surveys, such as patient comments and numerical ratings. PediPlace compared appointment lengths between July–December 2018 and January–June 2019, dividing the year into 2 parts before and after 2 of the QI interventions (revising MA and provider scripts and improving tracking). Each half consisted of approximately 10,000 appointments.

### Analysis

PediPlace analyzed over 2,000 patient satisfaction surveys. These surveys were from December 2017, April 2018, May–July 2018, September 2018, January–February 2019, May–June 2019, July 2019, and August 2019. The survey response rate was approximately 10%. Each patient received a paper survey form at the visit and had the opportunity to leave comments. PediPlace surveys were developed by the CEO with board input to gather patient feedback. The survey collected data on the ease of appointment scheduling, quality of provider, and overall satisfaction of care (**see Fig. 1, Supplemental Digital Content 1**, http://links.lww.com/PQ9/A174). A PediPlace board member coded comments as positive, negative, or neutral based on positive and negative word choices and phrases. Comments mentioning long wait times were deemed negative.

PediPlace tracked the total time spent in the clinic for different appointment types. The total appointment time started when the patient checked in at the front desk and ended when a patient checked out at the front desk. PediPlace analyzed total appointment lengths for over 20,000 appointments from July 2018 to June 2019. The clinic studied how different factors, such as patient age, patient ethnicity, provider, and time of year, impacted the total appointment length. Data analysis was conducted using Microsoft Excel (Microsoft, Redmond, Wash.). We also counted the number of patients with recorded appointment lengths greater than 3 hours (due to tracking errors) to determine patient tracking efficacy. This metric indicated how many errors in the recording of time intervals occurred. Total appointment lengths over 3 hours were considered outliers and counted to quantify tracking errors. However, these times were subsequently removed from data analysis and did not affect the analysis.

### Ethical Considerations

The focus of this project was quality improvement and not human subjects’ research. QI projects aim to improve a process through iterative design, whereas human subjects’ research answers a specific research question using a rigid protocol. The project posed no risks to patients, as PediPlace removed all patient identifiers and personal health information from data collection. Survey data also did not include any personal health information. The analysis was purely to utilize evidence-based interventions to look at improvement in appointment lengths and the patient experience. Thus, Institutional Review Board oversight was not required. PediPlace is not affiliated with any organizations with Institutional Review Board oversight responsibilities.

## RESULTS

### Survey Results

**Fig. 5.**
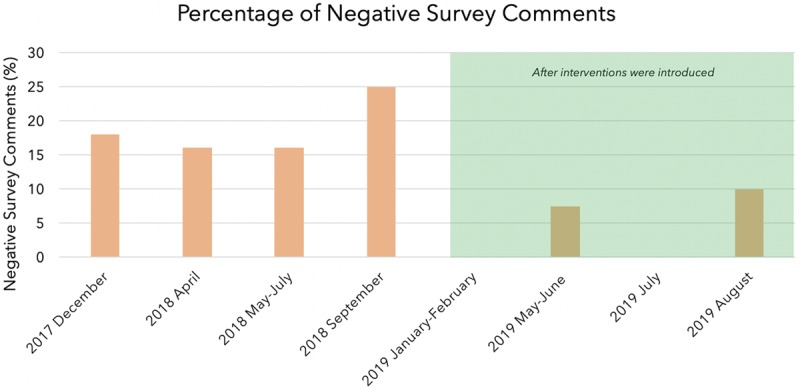
Graph showing the percentage of negative survey comments before and after interventions in January 2019.

Qualitative survey results showed an improvement in patient satisfaction from 2018 to 2019. Throughout late 2017 and 2018, of returned surveys, 16%–25% of survey comments were negative. After PediPlace implemented QI interventions at the start of 2019, 0% of comments were negative (Fig. 5). Data from May through August 2019 had a few negative comments, but the percentage of negative comments remained under 10% (Fig. 5). January 2019 surveys also included several positive comments regarding punctuality. One negative comment from August 2019 mentioned long wait times.

Quantitative survey ratings also suggested PediPlace patients were happier with their healthcare experience in 2019 compared to 2018. The “Overall Rating” question result in 2017 and 2018 was 3.8 out of 5; this measure rose in 2019 to 4.75 out of 5 in surveys from January to October 2019 (**see Fig. 2, Supplemental Digital Content 2**, http://links.lww.com/PQ9/A175). The question in 2019 was slightly modified from the previous year to read “Overall Quality of Care Today.”

In 2019, a new survey question asked patients to rate their wait time on a scale of 1–5 (1 = >30-minute wait, 5 = 5-minute wait). In January–October of 2019, patients rated PediPlace a 4.03, corresponding to approximately a 10-minute wait (**see Fig. 2, Supplemental Digital Content 2**, http://links.lww.com/PQ9/A175).

### Overall Appointment Length Results

**Fig. 6.**
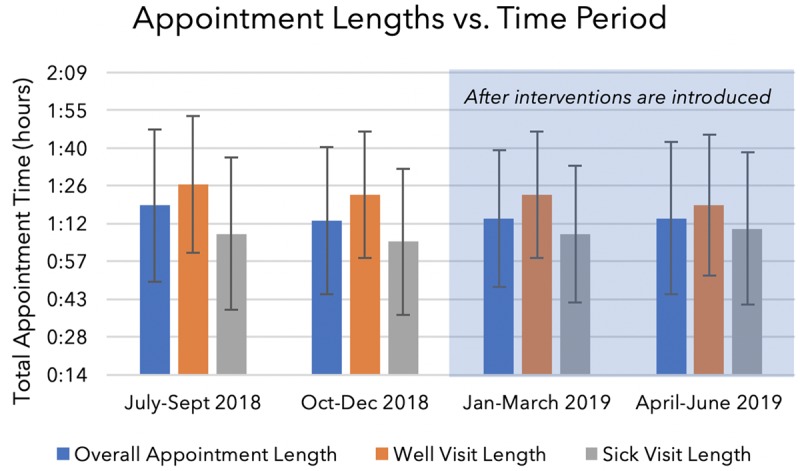
Bar graph showing the average appointment length for different appointment types before and after interventions in January 2019. Error bars indicate standard deviations in appointment lengths.

Overall appointment length did not change between July–September 2018 and March–June 2019. As seen in Figure 6, well visit appointment length decreased slightly from 1:27 to 1:19. However, sick-child appointment length increased rising from 1:08 to 1:10. Large standard deviations (ranging from 24 to 29 min) in the data indicated that these results were not significant. This finding suggested our interventions had no measurable impact on appointment lengths.

### Outliers

PediPlace counted the numbers of outliers (appointment lengths recorded as longer than 3 h) for each period. The outliers indicated poor patient tracking on the EHR interface. The outlier counts for July–September 2018 and October–December 2018 were 42 and 49, respectively. The outlier count dropped to 25 after the intervention in January 2019 to improve appointment tracking. However, the outlier count increased in the next quarter, April–June 2019, with 45 outliers.

### Hispanic/Latino Patients

We found no difference in average appointment length between Hispanic/Latino patients and other patient populations. Hispanic/Latino patients spent 1:14, and non-Hispanic/Latino patients spent 1:15 in the clinic. One July 2019 survey comment mentioned that the provider did not speak Spanish.

### Other Variables

PediPlace analyzed other factors potentially impacting appointment lengths. There was some variation between providers, but this did not affect appointment length. One nurse practitioner left between the 2 periods due to staff changes. Another provider was out of the office until June 2019, so data collected from the April–June 2019 period reflected only 1 month’s worth of data. For these reasons, we removed these 2 providers from data analysis. Patient age had no impact on total appointment length; young patients (ages 0–4) took 1:14 compared with school-aged patients (ages 5–12), and adolescents (ages 13 and up) who both took 1:15 on average.

## DISCUSSION

Extended wait and appointment lengths adversely affected patients and providers at PediPlace. A team effort aimed to reduce appointment lengths and provide activities for patients to improve their experience. This project demonstrated the importance of patient tracking, as recording errors can prevent valid data collection and misrepresent the situation. This QI initiative also highlighted the contributions of all clinic members, such as the front desk staff, MAs, and providers. The results suggested that patient satisfaction improved, whereas overall appointment length was unchanged. The initiatives were partially successful and provided insights for the next round of QI projects at PediPlace. These findings offer useful suggestions for other clinics, especially those serving pediatric patients.

Survey responses indicated improved patient satisfaction. Comparing 2018 with 2019 surveys, we found higher overall quantitative response ratings and fewer negative qualitative comments. However, the absolute number of comments in each quarter was quite low, so it is also possible the decrease in negative comments is due to chance. Although there was a slight wording change in the surveys from 2018 to 2019, PediPlace believes the data still indicated an improvement in patient satisfaction. These findings demonstrated that implementing QI initiatives correlated with better patient experience at PediPlace. Other factors may also have contributed to this improvement.

The clinic was unable to reduce sick-child visits to 45 minutes and well-child visits to 60 minutes. PediPlace found that the interventions did not impact overall appointment lengths. There was a slight decrease in well-child appointment lengths; this decline may be due to the elimination of repetition in MA and provider scripts, which only applied to well-child visits. However, there was a small increase in sick-child appointments. Large standard deviations indicated high variability in the data. The interventions had no significant impact on appointment length.

PediPlace did not examine time spent during each part of the visit, such as “time with provider” or “time waiting for immunizations.” This analysis is a potential area for future data collection because even a 5-minute difference in appointment length can alter patient perceptions of the healthcare experience. It is also possible that the wait times decreased, and more time was spent face-to-face with the provider. If this is the case, it is hard to discern by looking solely at total appointment length, and future projects examining each step of the appointment are necessary. With this data, PediPlace can analyze specific delays and develop targeted interventions. The new 2019 survey question regarding wait time will also provide further insights.

There was a substantial drop in outliers in the January–March 2019 quarter, immediately after the intervention to improve patient tracking. However, the outlier count increased in the following quarter, highlighting the need for continual reminders about patient tracking and checking out. The clinic now plans to include reminders at monthly meetings.

The data regarding Hispanic/Latino patients was unexpected because non-English speakers may have more difficulty communicating with providers and therefore have longer appointment lengths. The clinic viewed this result positively, as it indicated that the providers and staff are well equipped to work and communicate with their patient population. PediPlace attributed this result to almost all of the MAs being bilingual in English and Spanish. Some providers were also bilingual, and most had conversational Spanish competency. However, a July 2019 survey comment stated that the provider could not speak Spanish, indicating there is still room for improvement. Because the majority of the clinic’s patient population is Hispanic/Latino, it is important to ensure those language barriers are not a major problem. The project also found no differences in appointment lengths based on patient age.

### Limitations

Because PediPlace implemented the art activity kits in July 2019, it was difficult to obtain quantitative data on their success. However, anecdotal feedback from patients, families, and PediPlace staff at the time of submission of this QI report has been very positive. While these art activity kits were a useful initiative at PediPlace, this intervention may not apply to every clinic or patient population. The interventions in this project also were not implemented sequentially, making it difficult to discern individual impacts.

## CONCLUSIONS

By implementing a 3-part QI initiative, PediPlace improved the patient experience at the clinic. The initiative had no impact on appointment length. This project provided useful insights for the clinic to continue QI projects.

In the future, the clinic would like to continue monitoring survey responses and tracking appointment lengths. Over time, PediPlace hopes survey responses will shed light on the impact of the art activity kits, especially with regards to patient satisfaction. PediPlace will analyze all 2019 surveys and will include a question regarding art kits in the 2020 surveys. Gathering more feedback will help determine what other initiatives can benefit PediPlace. In the future, PediPlace plans to improve patient-provider communication by giving time estimates for each step of the appointment.

Furthermore, more precise appointment tracking will allow the clinic to quantify delays in each step of the appointment. PediPlace will continue implementing sustainable QI initiatives that benefit the clinic and patient population by reducing wait times while maintaining or increasing face-to-face time with providers.

## DISCLOSURE

The authors have no financial interest to declare in relation to the content of this article.

## ACKNOWLEDGMENTS

The authors of this study would like to thank PediPlace employees and staff for their contributions and making the QI project possible.

A summer 2019 Benenson Award in the Arts grant from Duke University provided funding for the art activity kits used in the initial implementation of this QI project.

## Supplementary Material


