# Supplementary material for: A Three-part Quality Improvement Initiative to Increase Patient Satisfaction and Reduce Appointment Time
Source: Pediatr Qual Saf. 2020 Mar 9;5(2):e277. doi: 10.1097/pq9.0000000000000277 (PMC7190244; doi:10.1097/pq9.0000000000000277)
Supplement: Supplementary file 1 [file pqs-5-e277-s001.pdf]

PediPlace
MAKING HEALTHCARE A REALITY FOR EVERY KID

1) How long has your child been a patient at PediPlace?
◊ First Visit ◊ 1 – 3 months ◊ 4 – 6 months ◊ 7 – 12 months ◊ 1 – 2 years ◊ Over 2 years

2) Since becoming a PediPlace patient, has the number of your emergency room visits:
◊ Decreased ◊ Increased ◊ Stayed the Same

3) Do you consider PediPlace to be your child's medical home?
◊ Yes ◊ No

4) How easy was if for you to make this appointment?
◊ My Appointment was made at my last visit
◊ Very Easy
◊ Somewhat easy
◊ It took awhile
◊ Difficult

5) The person at the front desk was friendly and helpful.
◊ Strongly agree ◊ Agree ◊ Neither ◊ Disagree ◊ Strongly Disagree

6) Are you and your children enrolled for the PediPlace Patient Portal?
◊ Yes ◊ No

7) Would you like to enroll in the PediPlace Patient Portal?
◊ Yes ◊ No

8) What was the reason for your visit today?
◊ Sick Visit ◊ Well Child Visit

9) If you answered sick above, how quickly were you able to schedule an appointment?
◊ In less than 3 hours ◊ Same day ◊ Next day
◊ In 2 days ◊ More than 2 days ◊ Does not apply

10) How long did you wait before being seen today?
◊ 5 Minutes ◊ 10 Minutes ◊ 20 minutes
◊ 30 minutes ◊ More than 30 minutes

11) Did the provider explain things in a way that was easy for you to understand?
◊ Yes ◊ No

12) How happy were you with the care given to you by the provider?
◊ Happy ◊ Unhappy

13) Overall how would you rate the quality of care you received today?
☆ ☆ ☆ ☆ ☆

14) Please use this space for any compliments, comments and/or concerns.

PediPlace
MAKING HEALTHCARE A REALITY FOR EVERY KID

1) Cuanto tiempo ha sido su hijo paciente de PediPlace?
◊ Primera Visita ◊ 1 – 3 meses ◊ 4 – 6 meses ◊ 7 – 12 meses ◊ 1 – 2 anos ◊ Mas de 2 anos

2) Desde que se convirtió en paciente de PediPlace, tiene el número de visitas a la sala de emergencias:
◊ Disminuido ◊ Aumentado ◊ Se mantuvo igual

3) Nos considera su proveedor de atencion primaria (o centro medico)?
◊ Si ◊ No

4) Que tan facil fue si para usted hacer esta cita?
◊ Mi cita fue hecha en mi ultima visita.
◊ Muy facil
◊ Algo facil
◊ Se tomo un tiempo
◊ Fue dificil

5) La persona en la recepcion era amable y servicial:
◊ Totalmente de acuerdo ◊ De acuerdo ◊ Ninguno ◊ Discrepar ◊ Muy en desacuerdo

6) Esta usted y sus hijos inscritos en el Portal para pacientes de PediPlace?
◊ Si ◊ No

7) Le gustaria inscribirse en el Portal de Pacientes PediPlace?
◊ Si ◊ No

8) Cual fue el motive de su visita hoy?
◊ Visita de enfermedad ◊ Bien vista del Nino

9) Si contest "enfermo" arriba, que tan rapido pudo programar una cita?
◊ Menos de 3 horas ◊ Mismo Dia ◊ Proximo Dia
◊ En 2 dias ◊ Mas de 2 dias ◊ No Aplicable

10) Cuanto tiempo espero antes de ser visto hoy?
◊ 5 Minutos ◊ 10 Minutos ◊ 20 minutos
◊ 30 minutos ◊ Mas de 30 minutos

11) El proveedor le explico las cosas de una manera que le resultaba facil de entender:
◊ Si ◊ No

12) Que tan feliz estuvo con la atencion que le brindo el proveedor?
◊ Contento ◊ No Feliz

13) En general, ¿Cómo calificaría la calidad de la atención que recibió hoy?
☆ ☆ ☆ ☆ ☆

14) Por favor utilice este espacio para cualquier queja, comentario o sugerencia.
