# Supplementary material for: A Three-part Quality Improvement Initiative to Increase Patient Satisfaction and Reduce Appointment Time
Source: Pediatr Qual Saf. 2020 Mar 9;5(2):e277. doi: 10.1097/pq9.0000000000000277 (PMC7190244; doi:10.1097/pq9.0000000000000277)
Supplement: Supplementary file 2 [file pqs-5-e277-s002.docx]

|  | **December 2017** | **April 2018** | **May-July 2018** | **September 2018** | **January-February 2019 (post-intervention)** | **May-June 2019** | **July 2019** | **August 2019** |
| --- | --- | --- | --- | --- | --- | --- | --- | --- |
| Number of comments | 11 | 25 | 18 | 8 | 16 | 27 | 13 | 20 |
| Number of negative comments | 2 | 4 | 3 | 2 | 0 | 2 | 0 | 2 |
| **Percentage of negative comments** | **18%** | **16%** | **16%** | **25%** | **0%** | **7.4%** | **0%** | **10%** |
| Number of survey responses | 162 | 231 | N/A | 181 | 212 | N/A* | N/A | N/A |
| **Overall Rating** (scale of 1-5)** | **4** | **3.65** | **N/A** | **3.66** | **4.75***** | **N/A** | **N/A** | **N/A** |

Supplementary figure 2. Table showing the percentage of negative survey comments and overall clinic ratings for each period. *There were 2294 survey responses from January-October 2019, but the numbers from each period were not available. ** In January 2019, PediPlace modified the overall rating question to "Quality of Care Today." ***The average rating from survey responses from January-October 2019 was 4.75, but average ratings from each period were not available. N/A indicates data was not available for the given period.
